# The use of hospital episode statistics (HES) records alongside clinical trials

**DOI:** 10.1186/1745-6215-16-S2-O28

**Published:** 2015-11-16

**Authors:** Belen Corbacho, Kerry Bell, Rita Santos, David Torgerson

**Affiliations:** 1 University of York, Department of Health Sciences, York, UK; 2 University of York, Centre of Health Economics, York, UK

## Introduction

Economic evaluation alongside trials is often based on data collected through self-reported questionnaires. Consequently, there are consistent problems with incomplete (missing) data irrespective of how well data collection methods are designed. Missing data poses a significant problem when conducting economic analyses of clinical trials, potentially leading to incorrect conclusions being drawn concerning an interventions’ cost-effectiveness.

## Trial design

Trials which collect economic data through multiple sources, such as hospital records in addition to patient self-reports, present an efficient and relatively simple means of investigating the impact different sources of data may have on the outcome of the economic evaluation of a trial. The present study uses data from a large randomised controlled trial of a family based intervention which collected health care resource use via both self-report, as well as through Hospital Episode Statistics (HES) data, sourced from the Health and Social Care Information Centre (HSCIC), to explore one method of dealing with missing data.

## Discussion

Multiple Imputation (MI) is the recommended statistical approach for evaluating the impact of missing data. Using data from the randomised controlled trial, we are able to compare two datasets; one derived via multiple imputation of self-reported data which contained missing values, and the other being HES data, which is assumed to be an accurate and objective representation of actual health care resource use.

## Conclusion

These data sets allow us to assess the effectiveness of multiple imputation at estimating the ‘truth’ and explore how the different methods of data collection can impact on final cost-effectiveness results.

